# 5-HT_7_ receptor signaling: improved therapeutic strategy in gut disorders

**DOI:** 10.3389/fnbeh.2014.00396

**Published:** 2014-12-11

**Authors:** Janice J. Kim, Waliul I. Khan

**Affiliations:** Department of Pathology and Molecular Medicine, Farncombe Family Digestive Health Research Institute, McMaster UniversityHamilton, ON, Canada

**Keywords:** inflammatory bowel disease, serotonin receptor type 7

## Abstract

Serotonin (5-hydroxytryptamine; 5-HT) is most commonly known for its role as a neurotransmitter in the central nervous system (CNS). However, the majority of the body’s 5-HT is produced in the gut by enterochromaffin (EC) cells. Alterations in 5-HT signaling have been associated with various gut disorders including inflammatory bowel disease (IBD), irritable bowel syndrome (IBS) and enteric infections. Recently, our studies have identified a key role for 5-HT in the pathogenesis of experimental colitis. 5-HT_7_ receptors are expressed in the gut and very recently, we have shown evidence of 5-HT_7_ receptor expression on intestinal immune cells and demonstrated a key role for 5-HT_7_ receptors in generation of experimental colitis. This review summarizes the key findings of these studies and provides a comprehensive overview of our current knowledge of the 5-HT_7_ receptor in terms of its pathophysiological relevance and therapeutic potential in intestinal inflammatory conditions, such as IBD.

## Introduction

The gastrointestinal (GI) tract contains an extensive system of endocrine cells that are interspersed amongst gut epithelial cells (Rehfeld, 1998). There are several subpopulations of endocrine cells, which release various biologically active compounds such as gastrin, secretin, cholecystokinin, chromogranin, and serotonin (5-hydroxytryptamine; 5-HT; Sharkey and Mawe, 2002). Enterochromaffin (EC) cells are the best characterized subset of enteric endocrine cells and constitute the largest endocrine cell population in the gut (Ham, 2002; Sharkey and Mawe, 2002; Ku et al., 2006; Gunawardene et al., 2011). EC cells arise from multi-potent stem cells located near the base of the crypts of Lieberkühn and are the body’s main source of 5-HT (Sharkey and Mawe, 2002; Gunawardene et al., 2011). Alterations in EC cells and 5-HT signaling has been shown to be associated in a number of GI disorders including inflammatory bowel disease (IBD), irritable bowel syndrome (IBS), and enteric infections, emphasizing the importance of 5-HT signaling in intestinal homeostasis.

5-HT mediates many GI functions, including secretion and peristalsis, by activating of a diverse range of 5-HT receptors (Mawe and Hoffman, 2013). To date, seven types of 5-HT receptors have been identified and amongst these, five are expressed within the GI tract. This paper reviews information on the most recently identified class of 5-HT receptors, 5-HT_7_, and its role in the GI tract with a focus on its implications in understanding the pathophysiology of intestinal disorders.

## 5-HT in the GI tract

5-HT is a highly conserved biogenic amine that is found in separate peripheral and central tissue pools that are distinctly regulated by two different rate-limiting enzymes, tryptophan hydroxylase (TpH) 1 and 2, respectively (Walther and Bader, 2003; Walther et al., 2003). TpH1 found in EC cells catalyzes the majority of 5-HT production in the body (roughly ~90%). TpH1 converts dietary L-tryptophan to 5-hydroxytryptophan (5-HTP), which is then converted to 5-HT by L-amino acid decarboxylase (Bertrand and Bertrand, 2010). Once synthesized, 5-HT is packaged into granules by the vesicular monoamine transporter 1 (VMAT1; Rindi et al., 2004; Schäfermeyer et al., 2004) and released mainly from granules stored near the basal border of the EC cell, though some studies have identified granules near the apical membrane (Nilsson et al., 1987). EC cells release 5-HT in response to various mechanical and chemical stimuli, including bacterial toxins, in a calcium-dependent manner (Racké et al., 1996; Mössner and Lesch, 1998). Once released, 5-HT participates in various gut functions, including secretion and peristalsis, by activation of a diverse range of 5-HT receptors located in the lamina propria (LP; Mawe and Hoffman, 2013). The actions of 5-HT are terminated by uptake by the serotonin reuptake transporter (SERT) into adjacent epithelial cells (Martel et al., 2003) and degradation by monoamine oxidase A (MAO_A_). SERT is also found in platelets and enteric neurons (Bertrand and Bertrand, 2010), and is the target for important therapeutic drugs such as fluoxetine and citalopram, members of the family of serotonin-selective reuptake inhibitors (SSRIs). SERT mediated reuptake of 5-HT can be partially replaced by the dopamine transporter (DAT) and organic cation transporter (OCT), albeit at a lower affinity than SERT (Chen et al., 2001).

## 5-HT signaling in intestinal inflammation

IBDs, Crohn’s disease (CD) and ulcerative colitis (UC), are serious chronic inflammatory conditions of the human bowel currently affecting approximately 1–2 million people in the US and Canada (Loftus, 2004). Although the pathogenesis of IBD remains unknown, it is a multifactorial disease that involves both genetic and environmental components. As such, IBD is considered to be an inappropriate immune response that occurs in genetically susceptible individuals as a result of a complex interaction between environmental factors, microbial factors, and the intestinal immune system (Bouma and Strober, 2003; Bernstein and Shanahan, 2008; Koloski et al., 2008; Arnett and Viney, 2010). During the past five decades, the frequency of IBD has increased rapidly in highly industrialized Western nations with Canada having one of the highest incidence rates of both UC and CD worldwide (Fedorak et al., 2010).

Alterations in 5-HT signaling have been observed in IBD (Ahonen et al., 1976; Belai et al., 1997; El-Salhy et al., 1997; Magro et al., 2002; Coates et al., 2004) and changes to EC cell numbers and 5-HT content have been associated with both UC and CD (Bishop et al., 1987; Belai et al., 1997; El-Salhy et al., 1997). Changes in 5-HT signaling have also been shown in various experimental models of intestinal inflammation, including tri-nitrobenzene sulphonic acid (TNBS), di-nitrobenzene sulphonic acid (DNBS), and dextran sulfate sodium (DSS; Oshima et al., 1999; Linden et al., 2005; Khan et al., 2006). In all of these models, EC cell numbers and 5-HT levels are increased. In addition, infection with either *Trichuris muris* or *Citrobacter rodentium*, leads to an increase in EC cells numbers and/or 5-HT release, further supporting a role for 5-HT in inflammatory states (O’Hara et al., 2006; Motomura et al., 2008).

5-HT itself also plays a key role in the generation of intestinal inflammation. Previously, we have shown that there is significant reduction in intestinal inflammation post- DSS and DNBS-induced colitis when intestinal 5-HT levels are reduced by genetic deletion of the rate-limiting TpH1 enzyme or by using parachlorophenylalanine (pCPA), while replenishing 5-HT levels intensifies colitis severity (Ghia et al., 2009). In turn, studies have also shown that chemical-induced colitis or spontaneous colitis associated with an IL-10 deficiency is increased in severity when coupled with the 5-HT enhancing effects of a knockout of SERT (Bischoff et al., 2009; Haub et al., 2010). Prior approaches aimed at blocking 5-HT synthesis by a pharmacological agent through inhibition of TpH, as with pCPA, have been impeded by adverse effects to brain 5-HT synthesis leading to alterations in central nervous system (CNS)-mediated functions (Ruhé et al., 2007). Recently, we have also shown that blocking 5-HT synthesis using an orally-delivered small molecule TpH inhibitor, telotristat etiprate (LX1032/LX1606), effectively reduces peripheral 5-HT synthesis and both chemical- and infection-induced intestinal inflammation (Kim et al., 2013b). This compound is unable to cross the blood-brain barrier (Savelieva et al., 2008) and does not appear to affect enteric neuronal TpH2 (Margolis et al., 2013). Oral administration of LX1606 significantly depletes intestinal 5-HT levels but does not affect brain 5-HT levels. Margolis et al. (2013) also evaluated the effect of LX1606 on neuronal 5-HT stores using immunocytochemical techniques and found that LX1606 did not affect the proportion of myenteric 5-HT-immunoreactive neurons or the area of myenteric plexus occupied by 5-HT-immunoreactive nerve fibers. This suggests that while LX1606 significantly depletes 5-HT stores from EC cells, entire neuronal 5-HT stores are maintained and therefore, LX1606 and similarly related peripheral TpH inhibitors appear to fail to enter the myenteric plexus and/or inhibit enteric neuronal TpH2.

The precise mechanisms by which 5-HT exerts its pro-inflammatory actions remains to be determined. To elucidate this mechanism, we assessed the role of 5-HT in dendritic cell (DC) function in relation to gut inflammation. EC cells are located in very close proximity to or in contact with immune cells such as DCs (Yang and Lackner, 2004) and studies from our lab and others have shown an important role for 5-HT in immune regulation and in turn, immune-mediated alteration of EC cells/5-HT signaling (Wang et al., 2007; Li et al., 2011; Shajib et al., 2013). DCs are professional antigen-presenting cells with the ability to initiate adaptive immune responses. Intestinal DCs reside in the LP as such, are able to continuously sample luminal contents. DCs play a critical role in orchestrating immune responses and have been shown to be important in the generation of intestinal inflammation (Lipscomb and Masten, 2002; Berndt et al., 2007). DCs isolated from TpH1 deficient mice following DSS administration release significantly less IL-12 compared with DCs isolated from wild-type mice (Li et al., 2011). Interestingly, when DCs isolated from TpH1 deficient mice are cultured in the presence of 5-HT, this restores IL-12 levels to those comparable to DCs from wild-type mice suggesting a role of 5-HT mediated activation of DCs. Furthermore, when 5-HT stimulated DCs are transferred back into TpH1 deficient mice, there is significant increase in colitis severity and this is associated with higher myeloperoxidase (MPO) activity and pro-inflammatory cytokine (IL-1β and IL-6) levels. This suggests that 5-HT mediated modulation of DC function is important in the pathogenesis of colitis though research targeting 5-HT signaling is needed to translate these observations for clinical utilization and to design a therapeutic strategy for colitis.

## Intestinal inflammation and the gut-brain-axis

The gut-brain-axis is a bi-directional neuro-humoral communication system that links gut and brain function in health and disease, and contributes to GI functions, including motility, secretion, visceral sensations, and mucosal immunity (Collins and Bercik, 2009; El Aidy et al., 2012; Forsythe and Kunze, 2013). The importance of this axis is demonstrated by its role in IBS and is reflected in the high prevalence of psychiatric morbidity in IBS (Whitehead et al., 2002). There is also growing evidence that the gut-brain-axis plays a role in IBD (Graff et al., 2009; Bonaz and Bernstein, 2013). IBD results in high morbidity and mortality and severely compromises quality of life and life expectancy. In recent years, there has been increasing recognition that depression can worsen the course of IBD (Mardini et al., 2004; Mittermaier et al., 2004; Persoons et al., 2005) and it has been shown that persons with IBD have higher rates of depression (in addition to panic, generalized anxiety, obsessive-compulsive disorders) compared with control populations (Walker et al., 2008). The mechanisms underlying this relationship in terms of cause-and-effect are currently unclear. In a study by Walker et al. (2008), it was reported that patients with IBD have a higher 12-month and also lifetime prevalence of major depression whereby approximately half experienced a first episode of depression more than 2 years before the onset of IBD. Depression may also negatively affect the course and outcome of disease. A prospective study by Mittermaier et al. (2004) found that in patients with IBD, those with significant depressive symptoms (at baseline) had relapses that occurred sooner and more frequently. In patients with CD, major depressive disorder has been reported as a risk factor for failure to achieve remission with infliximab treatment and an earlier need for retreatment (Persoons et al., 2005). In addition, studies have found that patients with active disease report higher levels of depression and anxiety while those with quiescent disease report lower levels (Porcelli et al., 1996; Levenstein, 2002; Larsson et al., 2008). As such, it has been proposed that treatments that improve mood may be useful in improving symptoms and disease activity in IBD. Antidepressants such as SSRIs are generally well tolerated and are successful in relieving psychological symptoms in about 30–40% of patients (Trivedi et al., 2006; Krishnan and Nestler, 2008). Recently, it was reported that antidepressants used to treat concomitant mood disorders in patients with IBD improves relapse rates and use of corticosteroids when compared with matched controls. Whether this occurred through a direct effect of the drug on the GI tract, or indirectly via improvement in mood and stress response was not investigated (Goodhand et al., 2012). Uncontrolled case report studies have also reported improvements not only in depression but also in IBD symptom scores (Mikocka-Walus et al., 2006). In contrast, data from a single open-label study of SSRI in IBD patients with depression reported improvements in depression but not in IBD activity (Walker et al., 1996). Moving forward, there is a need for randomized controlled trials to assess the effects of antidepressants such as SSRIs on disease activity in patients with IBD.

Studies using animal models have provided insight into mechanisms involved in the gut-brain-axis during GI inflammation and infection. Lyte et al. (1998) showed that mice treated orally with *Campylobacter jejuni* had increased anxiety-like behavior compared to saline-treated control mice. This was without any increase in inflammatory mediators and likely due to activation of vagal ascending pathways. In experimental models that result in increased GI inflammation, there are increases in anxiety-like behavior. In animal models of colitis, mice treated with DSS show increased anxiety-like behavior (Bercik et al., 2011). In addition, mice infected with *Trichuris muris* demonstrated intestinal inflammation that was associated with increased anxiety-like behavior when tested using the light/dark test and step-down test methods (Bercik et al., 2010). This was accompanied by decreased brain derived neurotropic factor (BDNF) expression in the hippocampus, and elevated levels of TNF-α, INF-γ, and kynurenine. Abnormal behavior (but not BDNF levels) was normalized by treatment with immunomodulators, etanercept and budesonide. Interestingly, both behavior and BDNF levels normalized following administration with probiotic *Bifidobacterium longum* suggesting a role for gut microbiota in modulating behavior. The role of the microbiota on the gut-brain-axis, however, is beyond the scope of this review, and has been extensively reviewed elsewhere (Cryan and O’Mahony, 2011; Collins et al., 2012). What is clear from the growing body of literature, is that the gut microbiome plays a critical role in regulating normal function of the gut-brain axis. Recently, there is a growing body of evidence looking at the role of 5-HT and the gut microbiome suggesting that 5-HT may be critically involved at every level of the brain-gut-microbiome axis (as reviewed by O’Mahony et al., 2014). With a better understanding of the interaction between this axis and the 5-HT system, this could aid in the design and development of novel therapeutic strategies for intestinal disorders that target 5-HT signaling with far-reaching effects beyond the gut. This may be particularly relevant in GI inflammatory disorders such as IBS and IBD with reported psychiatric comorbidities.

## 5-HT_7_ receptors in the gut

The discovery of 5-HT in the late 1940s was shortly followed by evidence for 5-HT receptor heterogeneity. To date, seven distinct families of 5-HT receptors have been identified, with some families consisting of various subpopulations (Hoyer et al., 2002). Five of the seven known families (5-HT_1_, 5-HT_2_, 5-HT_3_, 5-HT_4_, and 5-HT_7_ receptors) are expressed in the gut (Hoyer et al., 2002), with the 5-HT_3_ and 5-HT_4_ receptor subtypes being the most extensively studied. 5-HT_3_ and 5-HT_4_ receptors have been targeted for the treatment of diarrhea and constipation, respectively (Mawe and Hoffman, 2013). The 5-HT_7_ receptor is the most recently discovered member of the 5-HT receptor family and has since been cloned in rat (Lovenberg et al., 1993; Meyerhof et al., 1993; Ruat et al., 1993; Shen et al., 1993), mouse (Plassat et al., 1993), guinea pig (Tsou et al., 1994), porcine (Bhalla et al., 2002), and human (Bard et al., 1993).

The 5-HT_7_ receptor is expressed in both the CNS and in peripheral tissues. In the CNS, pharmacological and animal studies using 5-HT_7_ receptor deficient mice have established roles for the 5-HT_7_ receptor in control of circadian rhythms and thermoregulation (Lovenberg et al., 1993; Tsou et al., 1994; Hedlund et al., 2003), learning and memory (Roberts and Hedlund, 2012), and mood disorders including depression (Hedlund, 2009; Mnie-Filali et al., 2009). In the periphery, 5-HT_7_ receptors have been found to be expressed in the colon, ileum, and stomach with low expression in the spleen, liver, and kidney (Bard et al., 1993). 5-HT_7_ receptors have also been reported to be expressed on human enterocyte-like cell line, Caco-2 cells, and was found to modulate SERT activity (Iceta et al., 2009). Blood-derived DCs also express the 5-HT_7_ receptor (Shen et al., 1993; Vanhoenacker et al., 2000; Idzko et al., 2004).

**Figure 1:**
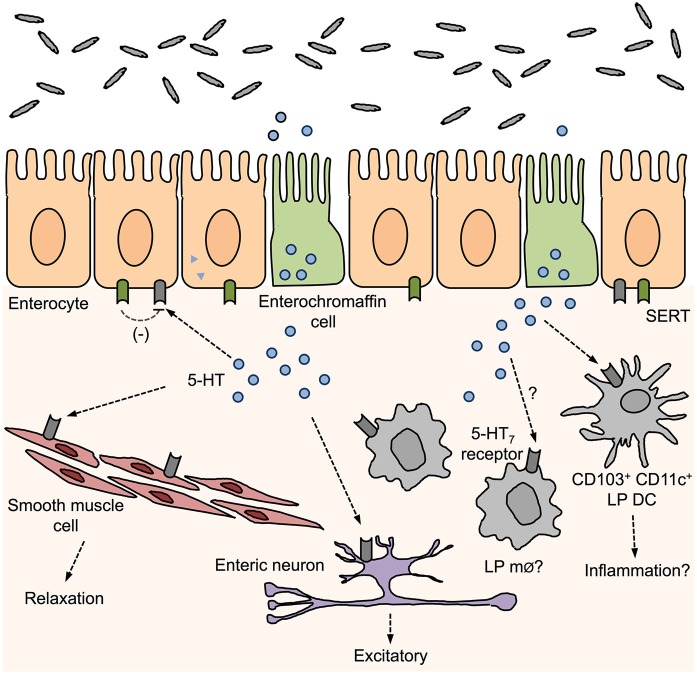
**Distribution of 5-HT_7_ receptors in the gut and proposed roles in gut function**. 5-hydroxytryptamine (5-HT) released from enterochromaffin cells can act on surrounding 5-HT_7_ receptors that are expressed by smooth muscle cells, enteric neurons, enterocytes, and immune cells. Activation of 5-HT_7_ receptors can influence muscle tone, enteric neuron excitation, and have been proposed to inhibit SERT activity and promote inflammation by activation of LP dendritic cells (DCs).

Specifically within the gut, 5-HT_7_ receptors are expressed on smooth muscle cells, enteric neurons, and within the solitary intestinal lymphoid tissue, small-sized intestinal lymphoid structures scattered through the small intestine (Tonini et al., 2005; Guseva et al., 2014). Recently, we have shown that the 5-HT_7_ receptor is also expressed on intestinal LP DCs (Kim et al., 2013a; Figure 1). DCs represent a heterogeneous population with functional diversity with different DC subsets having distinct sets of cell surface antigens. Although CD11c is the classical integrin marker used to distinguish DCs from macrophages (whereby CD11b^+^ CD11c^−^ and CD11b+/− CD11c^high^ are classified as macrophages and DCs, respectively), this becomes more difficult when distinguishing between LP macrophage and DC populations, as LP macrophages express both CD11b and CD11c markers (Mowat and Bain, 2011). Therefore, it is important to use differential expression of integrin CD103 (αE integrin) to reliably distinguish between these two populations. We found that isolated intestinal CD103^+^ CD11c^+^ cells were positive for 5-HT_7_ receptor expression whereas no significant amount was detected on CD103^−^ CD11c^+^ cells. Adding to this finding, Guseva et al. (2014) recently reported that CD11c+ CD86+ cells colocalize with 5-HT_7_ receptor staining in colon samples collected from both inflamed and non-inflamed areas of patients with CD. CD86 is a co-stimulatory molecule found on mature DCs. These findings suggest that 5-HT_7_ receptor expressed by DCs may play a role in modulating intestinal inflammation in this patient population.

Under physiological conditions, there is evidence that 5-HT_7_ receptors play a role in motility by mediating smooth muscle relaxation in colon (Prins et al., 1999; Tonini et al., 2005) and ileum smooth muscle (Carter et al., 1995). In addition, they are believed to have a role in initiating murine colonic migrating motor complexes (Dickson et al., 2010). 5-HT_7_ receptors may also have a role in inhibition of peristalsis by 5-HT (Tuladhar et al., 2003; Table 1).

**Table 1:** **Effects of 5-HT_7_ receptor activation or inactivation on gut function**.

Species/Region	5-HT_7_ activation/inhibition	Effect	Reference
Guinea pig ileum	Antagonist SB-269970	Restores 5-HT inhibited peristalsis	Tuladhar et al. (2003)
	Antagonist SB-269970	Inhibits 5-HT inhibited peristalsis (via enteric neurons)	Tonini et al. (2005)
	Agonist 8-OH-DPAT	Elicits relaxation	Carter et al. (1995)
Guinea pig mesentary	Antagonist SB-269970	Inhibits 5-HT inhibited constriction	Chan and von der Weid (2003)
Mouse colon	Antagonist SB-269970 Antagonist SB-258719	Inhibits spontaneous colonic migrating motor complexes	Dickson et al. (2010)
Canine stomach	Antagonist SB-269970	Inhibits 5-Carboxamidotryptamine (5-CT) induced gastric relaxation	Janssen et al. (2002)
Human colon	Antagonist SB-269970	Inhibits 5-HT induced relaxation of human colonic circular muscle	Irving et al. (2007)
	Antagonist mesulergine	Inhibits 5-HT induced relaxation of circular muscle	Prins et al. (1999)

## 5-HT_7_ receptor signaling in intestinal disorders

### Irritable bowel syndrome

A number of studies have reported altered 5-HT signaling activity in intestinal disorders. Therapeutic drugs to target selective modulation of 5-HT activity—including SSRIs, 5-HT_3_ and 5-HT_4_ antagonists and agonists, respectively—have been used in the treatment of functional GI disorders such as IBS (Gershon and Tack, 2007; Beattie and Smith, 2008). Some of these drugs, however, have been associated with unwanted side effects (Ladabaum, 2003; Cole et al., 2004) and thus, prompts the need for more studies on 5-HT and its receptors in GI pathology and pathophysiology. IBS is a functional bowel disorder in which abdominal pain and discomfort is associated with altered bowel habits. 5-HT plays a key role in regulating motor functions of the GI tract and studies have suggested that 5-HT_7_ receptors mediate smooth muscle relaxation, adding to the rationale for investigating 5-HT_7_ receptor ligands in IBS. In addition, they play a role in regulation of nociceptive pathways (Meuser et al., 2002) and thus, may be involved in the pathological mechanisms underlying visceral paresthesia seen in IBS. Zou et al. (2007) investigated the role of 5-HT_7_ receptors in the pathogenesis of IBS in a rodent model and found that 5-HT_7_ receptor expression was increased in the hippocampus, hypothalamus, and intestine (ileum and colon) of IBS groups as compared to controls and this was associated with higher cAMP levels at these sites. In addition, there is a high prevalence of comorbid depression and anxiety disorders in IBS patients (Andresen and Camilleri, 2006). As 5-HT_7_ receptors have been linked with depression (although its role in anxiety is currently inconclusive) (as reviewed by Hedlund, 2009), it may be an attractive potential therapeutic target for IBS with effects extending beyond the gut.

### Inflammatory bowel disease

The role of 5-HT_7_ receptors in IBD is far less studied. Very recently, however, Guseva et al. (2014) has reported that 5-HT_7_ receptor expression is increased in inflamed sections of CD patients. In addition, using experimental models of colitis, we have previously reported that 5-HT_7_ receptor levels are increased in the colon of mice post- DSS-induced colitis (Kim et al., 2013a). In addition, Guseva et al. (2014) also found an up-regulation in 5-HT_7_ receptor expression in cecum and rectum in DSS-treated animals compared with controls. Ours and other studies have also shown that the 5-HT_7_ receptor is expressed on DCs (Idzko et al., 2004; Kim et al., 2013a; Guseva et al., 2014). EC cells, the major producer of 5-HT in the gut, are located in close proximity to these cells and thus, it is likely that there is interplay between these two systems. We proposed that inhibiting 5-HT signaling by blocking 5-HT_7_ receptor function would lead to attenuation of immune cell activation and subsequent inflammation. Indeed, we found that blocking 5-HT signaling by using a selective 5-HT_7_ receptor antagonist (SB-269970) or by genetic deletion of this receptor alleviated intestinal inflammation in two separate chemical models of colitis (DSS and DNBS) (Kim et al., 2013a). This was indicated by lower macroscopic damage of the colon and less severe histopathological damage as compared to vehicle-treated controls. In turn, this was associated with a decrease in various pro-inflammatory markers including MPO and cytokines IL-1β, IL-6, and TNF-α. The beneficial effects of targeting said receptor was also seen in a chronic model of DSS-induced colitis. Furthermore, we identified a role for 5-HT_7_ receptor mediated cytokine release by mature DCs isolated from DSS-treated mice. Similar effects of 5-HT on cytokine secretion have been observed in monocyte-derived DCs (Müller et al., 2009). Importantly, it was recently reported that increased 5-HT_7_ receptor-positive cells in DSS-treated mice were also positive for CD11c, suggesting that there is increased amount of 5-HT_7_ receptor expressing DCs upon inflammation (Guseva et al., 2014).

By using chimeric mice that were reconstituted with bone marrow (BM) cells lacking 5-HT_7_ receptor expression, we found that wild-type recipients that received BM cells from 5-HT_7_ receptor deficient donors showed lower disease activity and less severe histopathological damage (Kim et al., 2013a). This suggests that 5-HT_7_ receptor activation on immune cells play a key role in mediating intestinal inflammation in experimental colitis. Contrary to our data, Guseva et al. (2014) found that pharmacological blockade or genetic deletion of the 5-HT_7_ receptor in DSS-induced colitis exacerbated colitis severity in mice. This may in large be due to the notable differences in experimental design and housing condition of animals. For instance, Guseva et al. (2014) administered the 5-HT_7_ receptor antagonist at a lower dosage (100 nM) for a shorter period of time, while we administered the antagonist throughout the duration of the DSS treatment at a much higher dosing range (20–80 mg/kg).

DCs are important players that orchestrate downstream immune responses from initial sampling of the luminal environment. As such, DCs play a crucial role in priming responses to Th1 and Th17 cells (important producers of IFN-*γ* and IL-17, respectively). We reported alterations in colonic IFN-*γ* and IL-17 levels in 5-HT_7_ receptor antagonist-treated mice post DSS-induced colitis suggesting that there may be effects on T-cell responses when 5-HT_7_ receptor signaling is disrupted *in vivo* (Kim et al., 2013a). Further investigation of the role of 5-HT_7_ receptor signaling in T-cell function and examination of the role of DCs and sequential T cell activation in the context of gut inflammation will be necessary in order to elucidate the downstream effects of targeting this receptor in intestinal inflammation. In addition, the role of 5-HT_7_ receptor activation in modulating other DC functions such as immune cell survival, remain to be determined. Recently, it has been reported that 5-HT stimulates human macrophage polarization through 5-HT_2B_ and 5-HT_7_ receptors pointing to 5-HT as a potential target for modulating macrophage polarization and the potential of targeting 5-HT_2B_ and 5-HT_7_ receptors as therapies against inflammatory pathologies (de las Casas-Engel et al., 2013). Future studies investigating the role of these receptors on LP macrophages in the context of colitis would be interesting to determine the specific roles the 5-HT_7_ receptor has on different cell types and subpopulations. Given the heterogeneity seen amongst immune cells in the intestine, it would not be surprising for 5-HT_7_ receptor activation to play distinct roles in different cell types.

### Enteric infections

Alteration in the number of EC cells and 5-HT levels have been observed in enteric infection (Bearcroft et al., 1996; Spiller et al., 2000; Turvill et al., 2000; Kordasti et al., 2004; Wheatcroft et al., 2005). Recently, we examined the role of the 5-HT_7_ receptor during enteric infection by using a murine model of large intestinal nematode infection, *Trichuris muris*. We found that expulsion of worms was significantly delayed in 5-HT_7_ receptor deficient mice after *T. muris* infection and this was accompanied by an attenuation of infection-induced colonic muscle contractility. There was reduction in IL-9 levels in 5-HT_7_ receptor deficient mice as compared to wild-type after infection (Kim et al., 2012). This suggests that the 5-HT_7_ receptor plays an important role in generation of infection-induced intestinal muscle contractility, worm expulsion, and modulation of immune responses in the context of host defense in enteric infection.

## Conclusions and future directions

There is now abundant evidence in favor of an important role of 5-HT signaling in various gut disorders including IBD, IBS, and enteric infections. In the past two decades, there have been significant advances in our understanding of the 5-HT_7_ receptor. Despite our best efforts, there are many unanswered questions and new avenues of research that still warrant investigation. Here, we highlighted the role of this receptor in the gut specifically in relation to inflammation and functional disorders; however, novel functions and roles for these receptors continue to emerge. Further elucidating the role of these receptors in intestinal function, and in intestinal pathologies and pathophysiology will help us to better understand the underlying mechanisms of various common intestinal disorders such as IBD and IBS, and will ultimately lead to the development of novel therapies. Further studies investigating 5-HT_7_ receptor signaling in human samples from IBD patients and targeted inhibition of 5-HT_7_ receptor function on mucosal DCs will be important in determining the role of the 5-HT_7_ receptor in intestinal inflammation and to translate *in vivo* findings.

## Conflict of interest statement

The authors declare that the research was conducted in the absence of any commercial or financial relationships that could be construed as a potential conflict of interest.
